# Case Report: Transvaginal specimen extraction following totally laparoscopic D2 distal gastrectomy for gastric cancer in a patient with situs inversus totalis: with video

**DOI:** 10.3389/fonc.2023.1189948

**Published:** 2023-05-23

**Authors:** Zeyu Li, Xiaolong Zhang, Lifei Tian, Zheng Liu, Xinhua Liao, Jian Qiu, Guorong Wang, Likun Yan, Xiaoqiang Wang, Xishan Wang, Ruiting Liu

**Affiliations:** ^1^ Department of General Surgery, Shaanxi Provincial People’s Hospital, Xi’an, Shaanxi, China; ^2^ Department of Colorectal Surgery, National Cancer Center/National Clinical Research Center for Cancer/Cancer Hospital, Chinese Academy of Medical Sciences and Peking Union Medical College, Beijing, China; ^3^ Department of General Surgery, The First Affiliated Hospital of Xi’an Jiaotong University, Xi’an, Shaanxi, China

**Keywords:** situs inversus totalis, gastric cancer, natural orifice specimen extraction surgery, laparoscope, minimally invasive

## Abstract

Because of its significant advantage of fast postoperative recovery, natural orifice specimen extraction surgery (NOSES) has attracted increasing attention worldwide. However, the NOSES in gastric cancer (GC) treatment still needs more clinical practice, especially for the rare anatomical anomaly. Situs inversus totalis (SIT) is a rare autosomal recessive anatomical anomaly with an incidence ranging between 1/8,000 and 1/25,000 births. We present a video of transvaginal specimen extraction following totally laparoscopic D2 distal gastrectomy performed in a 59-year-old woman known to have SIT. Preoperative investigations revealed that the patient had early GC at the antrum. A gastroscopy report from the local hospital showed signet-ring cell carcinoma. The preoperative computed tomography scan revealed irregular thickening of the gastric wall at the junction of the greater curvature and antrum without metastasis to the lymph nodes. In total, laparoscopic D2 distal gastrectomy was performed with transvaginal specimen extraction. Billroth II with Braun anastomosis was performed for reconstruction. The length of the operation was 240 min without intraoperative complications and with minimal blood loss of 50 ml. The patient was uneventfully discharged on postoperative Day 7. The final pathology confirmed signet-ring cell carcinoma confined to the mucosal muscle without metastasis in 16 lymph nodes. Transvaginal specimen extraction following totally laparoscopic D2 distal gastrectomy can be safely performed in patients with SIT and has similar surgical outcomes to usual laparoscopic gastrectomy.

## Introduction

Situs inversus totalis, most often diagnosed by radiographic examination, is a rare autosomal recessive anatomical anomaly with an incidence ranging between 1/8,000 and 1/25,000 births (1). In minimally invasive surgeries, surgical procedures for SIT patients are considered to be a great challenge to surgeons due to the transposition of abdominal and thoracic viscera (2). Different from traditional laparoscopic surgery, natural orifice specimen extraction surgery (NOSES) is a novel technique for removing the specimen from the abdominal cavity through the anus or vagina without an additional incision (3). With its great advantages, including less pain, lower analgesia requirements, faster recovery, shorter hospital stay, better cosmetic results, and lower incisional hernia rates, the concept of NOSES has been widely accepted (4). However, there has been no case described in the literature that combined laparoscopic distal gastrectomy and NOSES for SIT patients.

## Case presentation

**Figure 1:**
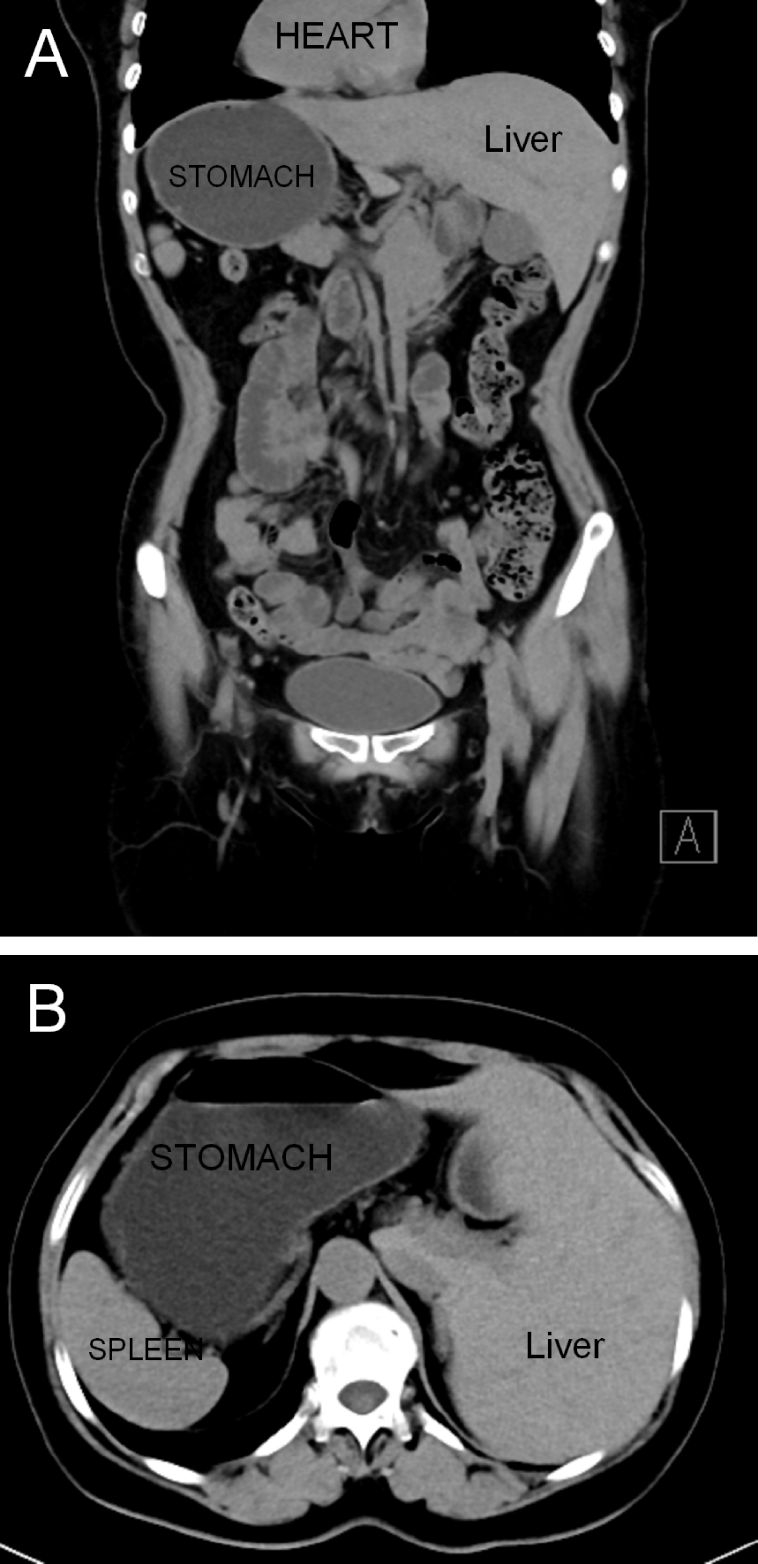
Abdominal computed tomography revealed a complete right–left reversal of the abdominal organs: **(A)** coronal view and **(B)** axial view.

A 59-year-old woman with a history of epigastric discomfort for more than 1 month was discovered to have SIT after diagnostic imaging was performed to investigate the cause of her symptoms. To further clarify the diagnosis, she underwent gastroscopy at a local hospital, and the results showed signet-ring cell carcinoma. The patient had no significant drug history or family history, and her physical examinations were unremarkable. The preoperative computed tomography scan revealed SIT in addition to irregular thickening of the gastric wall at the junction of the greater curvature and antrum without metastasis to the lymph nodes (**Figure 1**). The patient underwent hysterectomy at the local hospital because of hysteromyoma. After obtaining informed consent, laparoscopic distal gastrectomy with transvaginal NOSE was performed.

**Figure 2:**
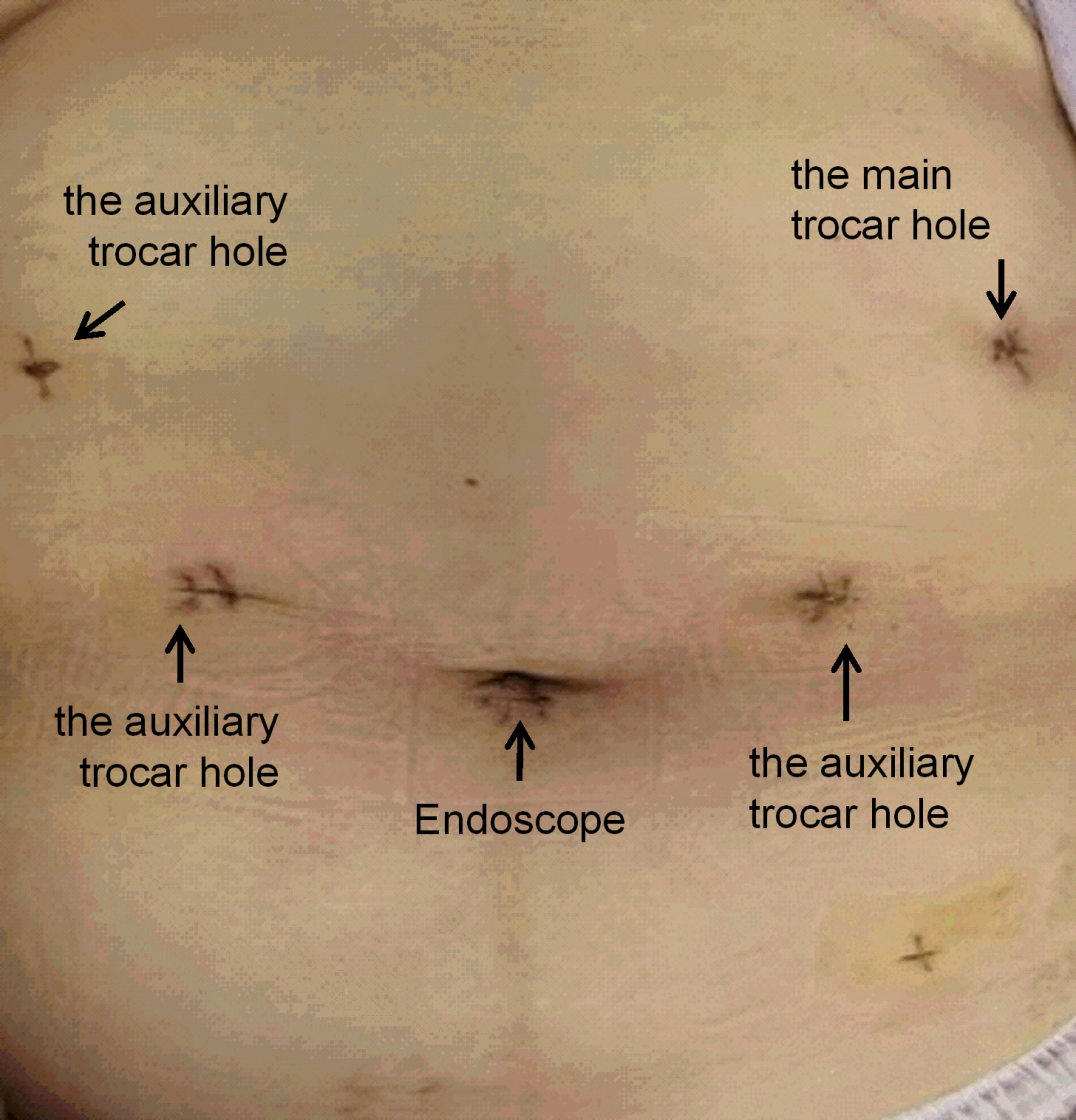
Port setup.

**Figure 3:**
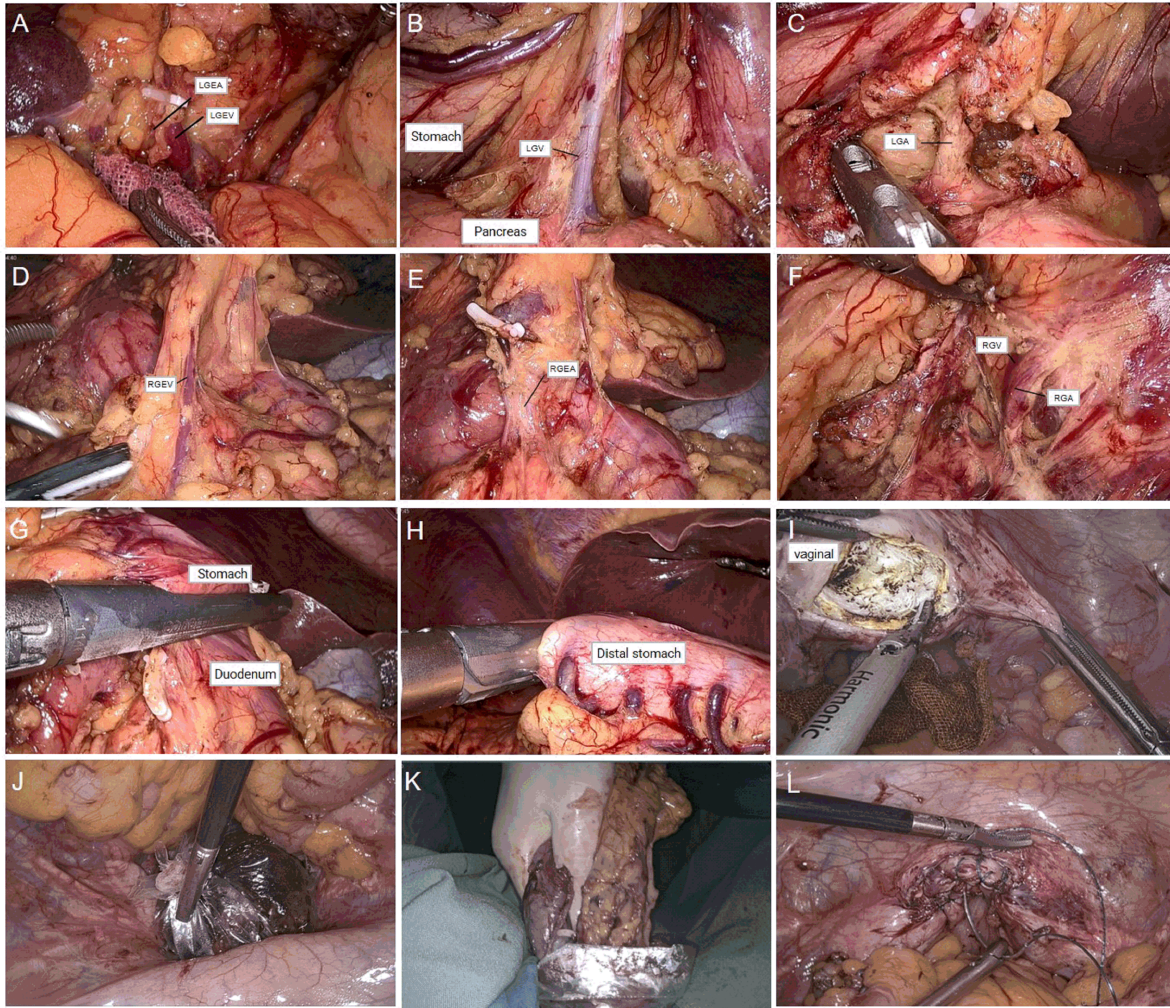
**(A)** Dissection of left gastroepiploic vessels. **(B, C)** Dissection of right gastroepiploic vessels. **(D, E)** Dissection of right gastric vessels. **(F)** Dissection of left gastric vessels. **(G)** Duodenal transection was performed using an endo linear stapler from the assistant port on the right abdomen. **(H)** Distal gastric transection was performed using an endo linear stapler from the assistant port on the right abdomen. **(I)** Incision of posterior vaginal wall. **(J, K)** Transvaginal specimen extraction. **(L)** Suture of the posterior vaginal wall.

The surgical procedures were as follows. After general anesthesia, a needle was placed in the umbilical hole, and the pneumoperitoneum pressure was set to 12 mmHg with CO_2_. A 12-mm trocar was placed under the costal margin of the left anterior axillary line as the main surgical hole, and a 5-mm trocar was placed 1 cm above the flat umbilicus of the left central clavicular line as the auxiliary surgical hole. Then, two 5-mm trocars were placed at the relative positions on the right side as auxiliary surgical holes (**Figure 2**). No implantation metastasis was found in the abdomen. The surgeon was located on the right side of the patient and successively dissected lymph node basins 6, 5, 8, 7, and 9. Then, the surgeon was positioned on the left side of the patient for the dissection of lymph node basins 12a, 12p, 1, and 3 in sequence. The left gastroepiploic artery and vein were ligated and clipped near the lower pole of the spleen. The right gastroepiploic artery and vein were separated, ligated, and clipped at the surface of the pancreatic head. The left and right gastric vessels were ligated and clipped in the same way (**Figure 3**). The duodenum and the gastric wall were intraperitoneally transected at an appropriate resection line using a linear stapling device. Billroth II with Braun anastomosis was performed for reconstruction.

**Figure 4:**
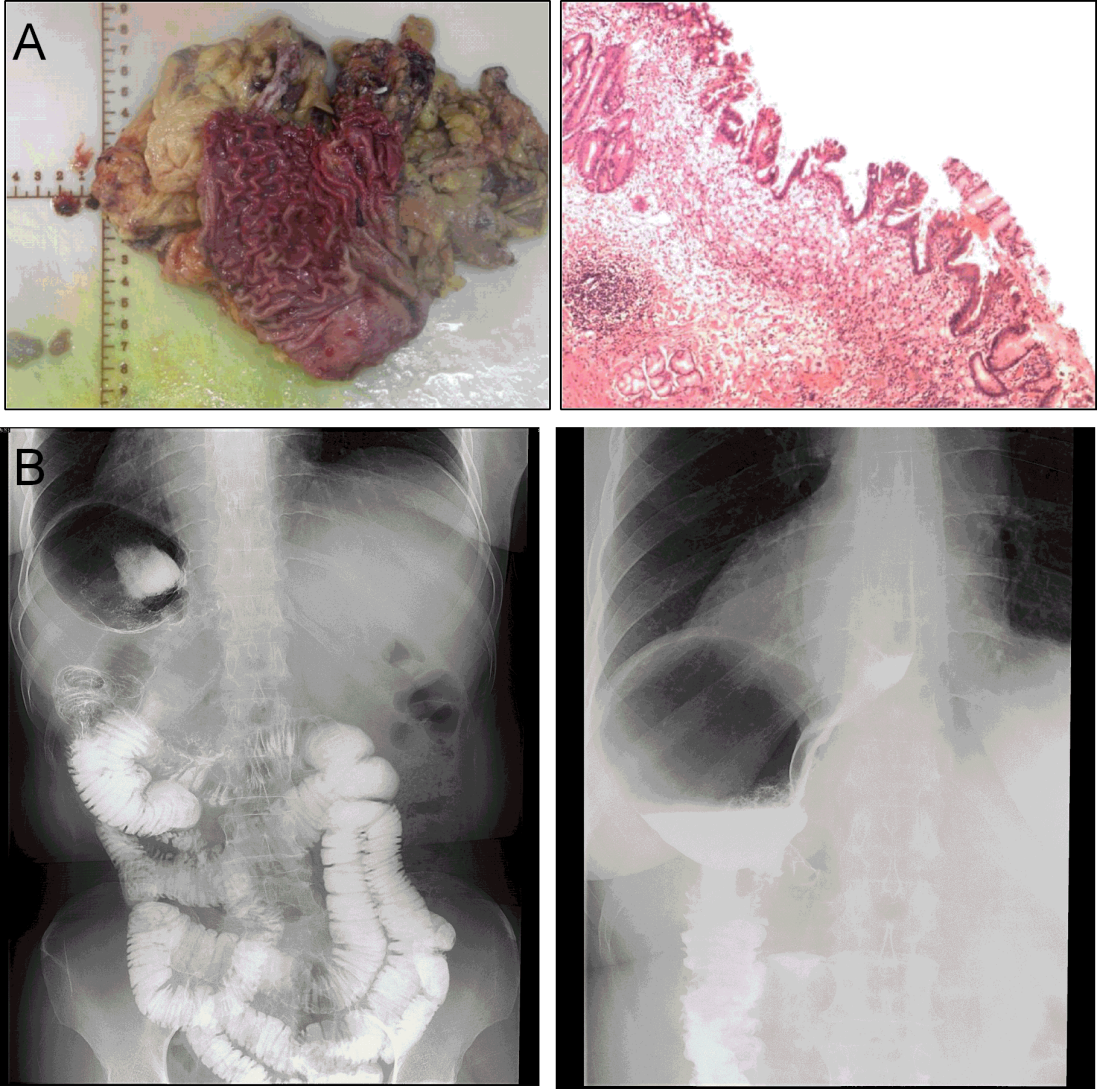
**(A)** Postoperative specimen and histology report. **(B)** The imaging of gastrointestinal tract showing no postoperative anastomotic complications at 7 days after operation.

For specimen extraction, the resected stomach and surrounding fatty tissue, including harvested lymph nodes, were placed in a plastic specimen bag. A 2-cm incision was made at the posterior vaginal wall, and the specimen bag was grasped by oval forceps and dragged out through the vagina. Then, the posterior vaginal fornix was closed with an absorbable suture. In this case, the length of the operation was 359 min, and the blood loss was 50 ml. The final pathology showed signet-ring cell carcinoma with invasion limited to the mucosal muscle (**Figure 4A**). No lymph node metastasis was found in the excised tissue, and the final stage was pT1aN0M0, Stage 1A. There were no complications after the operation, and the patient was discharged 7 days after surgery (**Figure 4B**).

## Discussion

The incidence rate of gastric cancer in China remains high, accounting for 40% of the world’s cases (5). All patients with operable disease should be recommended for radical resection combined with D2 lymphadenectomy (6). Laparoscopy-assisted gastrectomy (LAG) has been considered a standard surgical approach for the treatment of both early and advanced distal gastric cancer (7, 8). Although the incision is small, it has the risks of postoperative wound pain, infection, postoperative adhesion, and abdominal incision hernia. For this reason, surgeons need a more minimally invasive surgical technique. To solve the problems arising from small incisions, NOSES has been introduced as a less invasive surgery. Sang-Ho et al. first reported that four female patients with early gastric cancer received transvaginal specimen extraction following totally laparoscopic subtotal gastrectomy and achieved a satisfactory result (9). Sumer et al. also proved that totally laparoscopic subtotal gastrectomy with transvaginal specimen extraction is feasible in advanced gastric cancer (10). However, the NOSES in GC treatment still needs more clinical practice, especially for the rare anatomical anomaly.

SIT is an anatomical variation caused by the clockwise rotation of the embryonic midgut (11). When the fetus’ intrauterine bowel fails to rotate 270° anti-clockwise back into the abdomen, it exposes the small bowel to volvulus and ischemia of the bowel, which is a grave condition that has to be operated within hours (12). All of the thoracic and abdominal visceral organ positions in people with SIT are located conversely compared with normal people, similar to a mirror image of normal people. In 1936, the first SIT patient with gastric cancer was reported (6). In 2003, the first SIT patient with gastric cancer received treatment with laparoscopy-assisted gastrectomy (13). After that, several studies reported the difficulties of laparoscopic gastrectomy on SIT patients due to the difficult exposition of the surgical field and anatomical variation (14). Byoung et al. revealed that laparoscopic surgery for SIT patients with anatomical variations is a safe operative treatment if it can be offered with a careful approach and with great intraoperative attention to the inverted anatomical structures (15). However, there is no report about transvaginal specimen extraction following totally laparoscopic subtotal gastrectomy in SIT patients.

In our case, we offered a case in which the patient underwent laparoscopic D2 distal gastrectomy and was subjected to Billroth II with Braun anastomosis with transvaginal specimen extraction (GC-NOSES IV) (16). Although the patient had early gastric cancer (T1N0M0), the tumor cannot be extracted through the mouth. Meanwhile, the patient underwent a hysterectomy for uterine fibroids without postoperative pelvic inflammation or adhesion, which made the exposure of the surgical area more convenient even if she is a SIT patient. Therefore, we considered transvaginal specimen extraction to be feasible. Billroth II with Braun anastomosis was chosen because of its added advantages, such as a lower incidence of remnant gastritis, reflux esophagitis, dumping symptoms, and reflux symptoms. Moreover, we used the sterile protection devices when taking the specimen, thus avoiding over-pulling and compression of lesions during specimen extraction. In our case, the position of the surgeon and the assistant was the same as usual and the surgical procedure was completed successfully. Transvaginal specimen extraction following totally laparoscopic D2 distal gastrectomy can be safely performed in patients with SIT and has similar surgical outcomes to usual laparoscopic gastrectomy. We also provided the video of this surgery, and with the increased reporting of such cases, we can use artificial intelligence to help guide other surgeons with little or no experience with such condition (17).

## Conclusions

Gastric cancer patients with SIT are very rare; therefore, not much has been established in regard to the ideal surgical procedure. Transvaginal specimen extraction following totally laparoscopic D2 distal gastrectomy for SIT patients is feasible and safe, especially for early-stage patients.

## Data availability statement

The original contributions presented in the study are included in the article/Supplementary Material. Further inquiries can be directed to the corresponding author.

## Ethics statement

This study was approved by the Ethics Committee of Shaanxi Provincial People’s Hospital and strictly adhered to the tenets of the Declaration of Helsinki (Code of Ethical Approval for Scientific Research Project: 2019 Ethical Scientific Research Approval No. 2021-K200). All patients or their authorized representatives gave written informed consent before the operation. Written informed consent was obtained from the individual(s) for the publication of any potentially identifiable images or data included in this article.

## Author contributions

ZYL, XZ, and LT collected the patient’s clinical data. ZL, XL, JQ, GW, and LY analyzed and interpreted the data. XQW and XSW participated in revision. RL wrote the paper with input from all authors. All authors contributed to the article and approved the submitted version.

## References

[1] Gentile BA Tighe DA . Situs inversus totalis. N Engl J Med (2019) 380(24):e45. doi: 10.1056/NEJMicm1811002 31189040

[2] Alhossaini R Hyung WJ . Robotic assisted distal gastrectomy for gastric cancer in a patient with situs inversus totalis: with video. J Gastrointest Surg (2017) 21(12):2144–5. doi: 10.1007/s11605-017-3576-x 28900793

[3] Guan X Liu Z Longo A Cai JC Tzu-Liang Chen W Chen LC . International consensus on natural orifice specimen extraction surgery (NOSES) for colorectal cancer. Gastroenterol Rep (Oxf) (2019) 7(1):24–31. doi: 10.1093/gastro/goy055 30792863 PMC6375350

[4] Wolthuis AM de Buck van Overstraeten A D'Hoore A . Laparoscopic natural orifice specimen extraction-colectomy: a systematic review. World J Gastroenterol (2014) 20(36):12981–92. doi: 10.3748/wjg.v20.i36.12981 PMC4177477 25278692

[5] Yang L Kartsonaki C Yao P de Martel C Plummer M Chapman D . The relative and attributable risks of cardia and non-cardia gastric cancer associated with helicobacter pylori infection in China: a case-cohort study. Lancet Public Health (2021) 6(12):e888–e96. doi: 10.1016/S2468-2667(21)00164-X PMC8646857 34838195

[6] Wohnrath DR Araujo RLC . D2 lymphadenectomy for gastric cancer as an independent prognostic factor of 10-year overall survival. Eur J Surg Oncol (2019) 45(3):446–53. doi: 10.1016/j.ejso.2018.10.538 30392746

[7] Ding J Liao GQ Liu HL Liu S Tang J . Meta-analysis of laparoscopy-assisted distal gastrectomy with D2 lymph node dissection for gastric cancer. J Surg Oncol (2012) 105(3):297–303. doi: 10.1002/jso.22098 21952834

[8] Zhang CD Yamashita H Zhang S Seto Y . Reevaluation of laparoscopic versus open distal gastrectomy for early gastric cancer in Asia: a meta-analysis of randomized controlled trials. Int J Surg (2018) 56:31–43. doi: 10.1016/j.ijsu.2018.05.733 29860125

[9] Jeong SH Lee YJ Choi WJ Paik WY Jeong CY Park ST . Trans-vaginal specimen extraction following totally laparoscopic subtotal gastrectomy in early gastric cancer. Gastric Cancer (2011) 14(1):91–6. doi: 10.1007/s10120-011-0006-8 21264485

[10] Sumer F Kayaalp C Ertugrul I Yagci MA Karagul S . Total laparoscopic subtotal gastrectomy with transvaginal specimen extraction is feasible in advanced gastric cancer. Int J Surg Case Rep (2015) 16:56–8. doi: 10.1016/j.ijscr.2015.08.043 PMC4643334 26413924

[11] Robinson P . Situs inversus: when an incidental finding is not so incidental. J Paediatr Child Health (2017) 53(7):715–6. doi: 10.1111/jpc.13591 28670800

[12] Azzam A Abdulkarim AN Shehata AEM Mahran I Arafa A Arafat A . A report of two infant cases operated for jejunal duplication cyst associated with malrotation and volvulus. Int J Surg Case Rep (2020) 67:227–30. doi: 10.1016/j.ijscr.2020.02.009 PMC7047138 32113129

[13] Cao Y Li J Shen L Wang J Xia Z Tao K . Gastric cancer in a situs inversus totalis patient with multiple intestinal and vessel variations related to gastrectomy surgery: a case report and literature review. Med (Baltimore) (2017) 96(39):e8209. doi: 10.1097/MD.0000000000008209 PMC5626328 28953685

[14] Futawatari N Kikuchi S Moriya H Katada N Sakuramoto S Watanabe M . Laparoscopy-assisted distal gastrectomy for early gastric cancer with complete situs inversus: report of a case. Surg Today (2010) 40(1):64–7. doi: 10.1007/s00595-009-4007-8 20037843

[15] Suh BJ. A case of gastric cancer with situs inversus totalis. Case Rep Oncol (2017) 10(1):130–5. doi: 10.1159/000456539 PMC5301127 28203176

[16] Guan X Liu Z Parvaiz A Longo A Saklani A Shafik AA . International consensus on natural orifice specimen extraction surgery (NOSES) for gastric cancer (2019). Gastroenterol Rep (Oxf) (2020) 8(1):5–10. doi: 10.1093/gastro/goz067 32104581 PMC7034234

[17] Heba T Grasso V Tawfik S Gumbs A . The challenges of deep learning in artificial intelligence and autonomous actions in surgery: a literature review. Art Int Surg (2022) 2:144–58. doi: 10.20517/ais.2022.11

